# Artificial Waterbodies: A Valuable Source of eDNA for Detecting Threatened Birds

**DOI:** 10.1002/ece3.71509

**Published:** 2025-06-05

**Authors:** Gary Young, Benjamin L. Allen, Peter J. Murray, Elise M. Furlan

**Affiliations:** ^1^ Institute for Life Sciences and the Environment University of Southern Queensland Toowoomba Queensland Australia; ^2^ Department of Environment and Science Queensland Parks and Wildlife Service Brisbane Queensland Australia; ^3^ Centre for African Conservation Ecology Nelson Mandela University Port Elizabeth South Africa; ^4^ School of Agriculture and Environmental Science University of Southern Queensland Toowoomba Queensland Australia; ^5^ Centre for Conservation Ecology and Genomics, Institute for Applied Ecology University of Canberra Bruce Australian Capital Territory Australia

**Keywords:** eDNA, genetics, ground‐nesting bird, pigeon, threatened fauna, waterpoint

## Abstract

Environmental DNA (eDNA) has transformed biodiversity monitoring, especially in aquatic environments; yet, its application in terrestrial habitats remains limited. In arid regions, artificial waterbodies, such as farm dams and water troughs, serve as essential resources for wildlife and offer a promising but underutilised opportunity for eDNA‐based detection. Here, we designed and validated a highly sensitive, species‐specific quantitative PCR (qPCR) assay to detect the nationally threatened southern squatter pigeon ( *Geophaps scripta scripta* ). We validated the qPCR assay in the field by successfully detecting the target species at extremely low DNA concentrations (1 × 10^−7^ ng/μL; *r* ^2^ = 0.992) using both active syringe and passive filtration methods across multiple farm dams and water troughs on a 20,000‐ha cattle property in northern Australia. To complement eDNA analysis, we also undertook standardised 20‐min, 2‐ha bird surveys at these sites. Positive detections were recorded at both trough and dam sites during the austral tropical dry season. Notably, whilst eDNA detections and visual bird counts aligned in terms of the number of occupied sites, their exact locations did not always coincide, highlighting the complementary nature of these two monitoring techniques. This assay represents a significant advancement in the conservation of this threatened ground‐nesting species, demonstrating that eDNA sampling at artificial waterpoints is a viable tool for monitoring terrestrial fauna in remote, semi‐arid landscapes.

## 1 Introduction

Environmental DNA (eDNA) has emerged as a revolutionary tool for monitoring biodiversity, enabling the non‐invasive survey and detection of species through the identification of genetic materials (DNA or RNA) from substrates such as soil, air or water (Allen et al. 2023; Andersen et al. 2012; Johnson et al. 2023; Rees et al. 2014; Thomsen et al. 2012). eDNA surveys can be completed using metabarcode markers to detect the presence of multiple species (e.g., Thomsen et al. 2012) or by using species‐specific markers to detect the presence of a single species (e.g., Ficetola et al. 2008). Whilst metabarcoding can be a powerful approach to broadscale biodiversity monitoring, species‐specific eDNA surveys demonstrably provide a highly sensitive and cost‐effective alternative to traditional survey methods to infer the distribution of invasive (Hinlo et al. 2017; Smart et al. 2015), cryptic (Campbell et al. 2022) and threatened species (Day et al. 2019; Fediajevaite et al. 2021). The application of species‐specific eDNA monitoring is highly effective in freshwater and marine environments, with the majority of studies focused on large aquatic ecosystems (i.e., rivers, lakes and oceans) to detect aquatic species, particularly fish (Beng and Corlett 2020; Huang et al. 2022; Takahashi et al. 2023). Despite the extensive application of eDNA technologies to detect aquatic and, to a lesser extent, semi‐aquatic species, the use of eDNA for monitoring terrestrial vertebrates remains comparatively limited.

Small and localised sources of water, both natural and artificial, play an essential role in supporting a diverse range of terrestrial mammals, reptiles and birds, particularly in arid and semi‐arid areas (Abdu et al. 2018; Buono et al. 2019; Garcia‐Gonzalez and Garcia‐Vazquez 2011; Lisóon and Calvo 2014; Littlefair et al. 2024; Nieman and Leslie 2024; Zamora‐Marín et al. 2021). In Australia, estimates indicate at least 1.7 million farm dams (Malerba et al. 2021) along with countless water troughs (Brainwood and Burgin 2009) providing a vital resource for water‐dependent species whilst offering potentially valuable sites to target eDNA surveys for co‐occurring species. Whilst some studies have used eDNA to detect terrestrial species from small, natural waterbodies (Furlan et al. 2020; Michael et al. 2024), reports of eDNA surveys at artificial livestock waterpoints like farm dams or water troughs are scarce. A notable exception is McDonald et al. (2023), who detected higher rates of vertebrate taxa in eDNA samples from cattle troughs compared to natural waterbodies in a semi‐arid habitat. Similarly, Zamora‐Marín et al. (2021) revealed that livestock water troughs supported greater terrestrial bird species richness over larger‐sized artificial pools and ponds, underscoring the significance of these small, isolated habitats for terrestrial birds in semi‐arid landscapes.

Ground‐nesting birds are amongst the most threatened avian groups worldwide (Lees et al. 2022; Reif et al. 2023; Rosenberg et al. 2019). These species provide vital functional roles as seed‐dispersers and ecosystem engineers (Mariyappan et al. 2023; Whelan et al. 2008), yet species within the family Columbidae (pigeons and doves) face a disproportionately high risk of extinction relative to other avian families (Cambrone et al. 2023; Szabo et al. 2012; Walker 2007). Amongst these, the squatter pigeon (190–250 g *Geophaps scripta* , Temminck, 1821), a granivorous, ground‐nesting species endemic to eastern Australia (Crome 1976; Ward et al. 2021), may benefit from conservation efforts using eDNA surveys at remote waterpoints. The species is highly dependent upon and typically associated with water sources. Consequently, remote waterpoints (both natural and artificial) provide a vital resource for this species and represent potential hotspots for detecting squatter pigeon DNA. *G. scripta* prefer open eucalyptus woodlands with grassy understories on sandy soils and are distributed from New South Wales to Queensland's Cape York Peninsula (Frith 1982). Hybridisation occurs between the two subspecies: the common northern subspecies, *G. s. peninsulae*, and the threatened southern subspecies, *G. s. scripta*, where their ranges overlap along the Burdekin‐Lynd Divide of north Queensland (Ford 1986; Forshaw and Cooper 2015). The southern subspecies is poorly researched and monitored, and most sightings arise from ad hoc observations (Ward et al. 2021).

Populations of *G. s. scripta* have steadily declined since the early 19th century (Bennett 1891; Woinarski and Catterall 2004), with their range contracting markedly across New South Wales and southern Queensland (Franklin 1999; Smith et al. 1994; TSSC 2015). The subspecies is currently listed as Vulnerable under both Australian Commonwealth and Queensland legislation and Critically Endangered in New South Wales, largely due to sustained threats of habitat loss (Neldner et al. 2017), habitat degradation by overgrazing (Franklin 1999) and predation by invasive species (Woinarski et al. 2017). Efforts to detect *G. s. scripta* are hampered by their fragmented subpopulations and nomadic nature, possibly a result of historic changes and dependence on forage availability (Crome 1976; Garnett et al. 1998). Although there is no known seasonal variation in the species' activity patterns, eDNA detection rates may vary seasonally due to environmental factors, with seasonal variation in eDNA detectability commonly observed (e.g., Buxton et al. 2018; Furlan et al. 2016; Troth et al. 2021). During the dry season, as ambient temperatures rise and natural water sources dwindle, artificial livestock waterpoints in arid habitats may serve as vital DNA sinks, providing strategic sites to target eDNA surveys for *G. s. scripta*, enhancing eDNA detectability during the late dry season.

In this study, we aimed to (1) develop a species‐specific DNA assay to detect the southern squatter pigeon and (2) evaluate the assay's effectiveness in detecting eDNA from multiple water troughs and farm dams during the Australian tropical dry season. Our overall objective was to determine whether eDNA samples from these remote waterpoints could reliably confirm the presence of squatter pigeons. Using multiple eDNA sampling methods (active and passive filtration), we assessed the potential of these artificial waterpoints as focal sites to target eDNA surveys. Our findings demonstrate the utility of eDNA methods to detect and monitor threatened species in highly turbid environments and their potential in underexplored terrestrial habitats such as agricultural landscapes.

## 2 Materials and Methods

### 2.1 Quantitative PCR (qPCR) Assay Design

Tissue samples from the target species, *G. scripta* , and co‐occurring, related Columbidae (pigeons and doves) species were first obtained from the Queensland Museum and Museums Victoria (Table 1). A subsample of tissue was extracted using the Qiagen DNeasy Blood and Tissue kit (Qiagen Pty Ltd., Vic., Australia) following the manufacturer's protocol with a final elution to 100 μL in AE Buffer.

**TABLE 1:** List of target and co‐occurring Columbidae species with available tissue samples and/or sequence information. Location and year of tissue sample collection also provided.

Species	Common name	Tissue sample	Location	Year	Source	Voucher code/accession number
*Geophaps scripta scripta*	Squatter pigeon, southern sub‐species	Yes	Tambo Qld	2015	Queensland Museum	O33435
*Geophaps scripta scripta*	Squatter pigeon, southern sub‐species	Yes	Cathu State Forest Qld	1988	Museums Victoria (Ian Potter Collection)	Z50116
*Geophaps scripta scripta*	Squatter pigeon, southern sub‐species	Yes	Rockhampton Qld	1988	Museums Victoria (Ian Potter Collection)	Z50080
*Geophaps scripta peninsulae*	Squatter pigeon, northern sub‐species	Yes	Chillagoe Qld	2017	Queensland Museum	O33544
*Geopelia cuneata*	Diamond dove	Yes	Welford National Park Qld	2013	Queensland Museum	O33032
*Geopelia cuneata*	Diamond dove	No			GenBank	AF483280
*Geopelia humeralis*	Bar‐shouldered dove	Yes	Killarney Qld	2016	Queensland Museum	O33673
*Geopelia placida*	Peaceful dove	Yes	Noonbah Station Qld	2013	Queensland Museum	O33482
*Ocyphaps lophotes*	Crested pigeon	Yes	Noonbah Station Qld	Unknown	Queensland Museum	A008037
*Ocyphaps lophotes*	Crested pigeon	No			GenBank	Oclop11, EF373300.1, AF483286.1
*Phaps chalcoptera*	Common bronzewing	Yes	Noonbah Qld	1999	Queensland Museum	NA A009224
*Phaps chalcoptera*	Common bronzewing	No			GenBank	EF373316.1, AF483287.1
*Streptopelia chinensis*	Spotted turtle‐dove	No			GenBank	KP273832.1, AF483304.1, KP636801.1

All DNA extracts underwent PCR amplification to obtain sequence information targeting a 436 bp region of the 12S mitochondrial DNA gene using newly designed primers (forward primer: 5′‐CAAACTGGGATTAGATACCCCACTAT‐3′, reverse primer: 5′‐GAGGGTGACGGGCGGTATGT‐3′, modified from Fuller et al. 1998). Reactions took place on a CFX Connect Real‐Time PCR Detection System (Bio‐Rad) in 20 μL reaction volumes containing 1X MyTaq Red Mix (Bioline), 0.8 μM each of the forward and reverse primer and 2 μL DNA. Cycling conditions were 94°C for 2 min and 45 cycles of 94°C (30 s), primer annealing at 55°C (1 min) and extension at 72°C (30 s), with a final extension at 72°C (10 min).

PCR amplicons were purified and sent to the Biomedical Research Facility at the Australian National University for Sanger sequencing on an ABI 3730xl DNA Analyser using the above primers. Sequences were imported into Geneious 2022.2.2 (www.geneious.com) and edited manually. All available online sequences of closely related and co‐occurring species were obtained from GenBank (Benson et al. 2009) and were also imported into Geneious 2022.2.2 and co‐aligned.

Species‐specific primers were designed to target the squatter pigeon in a qPCR assay by identifying two approximately 20 bp regions lying ~80–200 bp apart that were conserved amongst squatter pigeons and contained sequence mismatches with co‐occurring species. Due to potential overlap in the range of the northern and southern subspecies, we attempted to design primers to specifically target the southern subspecies, *G. s. scripta*, despite high sequence similarity with the northern subspecies, *G. s. peninsulae*.

### 2.2 qPCR Assay Sensitivity and Specificity

Assay sensitivity was determined by performing qPCRs on positive eDNA from the target species in 12 replicate concentrations from 1 × 10^−1^ to 1 × 10^−7^ ng/μL. qPCRs were run in 20 μL reactions containing 1x TaqPathPro, 8 μM each of the forward and reverse primer, 0.15X SYBR Green Mix and 4 μL of DNA. Cycling conditions for the squatter pigeon assay were 95°C for 10 min and 50 cycles of 95°C (15 s) and primer annealing at 59°C (45 s), followed by a melt curve of 95°C for 15 s and step‐down to 60°C.

The assay was tested for specificity against both subspecies of the target species (i.e., *G. s. scripta* and *G. s. peninsulae*), as well as non‐target but potentially co‐occurring species *Ocyphaps lophotes* (crested pigeon), *Geopelia cuneata* (diamond dove), *G. placida* (peaceful dove), *Phaps chalcoptera* (common bronzewing) and *G. humeralis* (bar‐shouldered dove) in qPCR reactions as detailed above.

### 2.3 Study Site and eDNA Sampling

**FIGURE 1:**
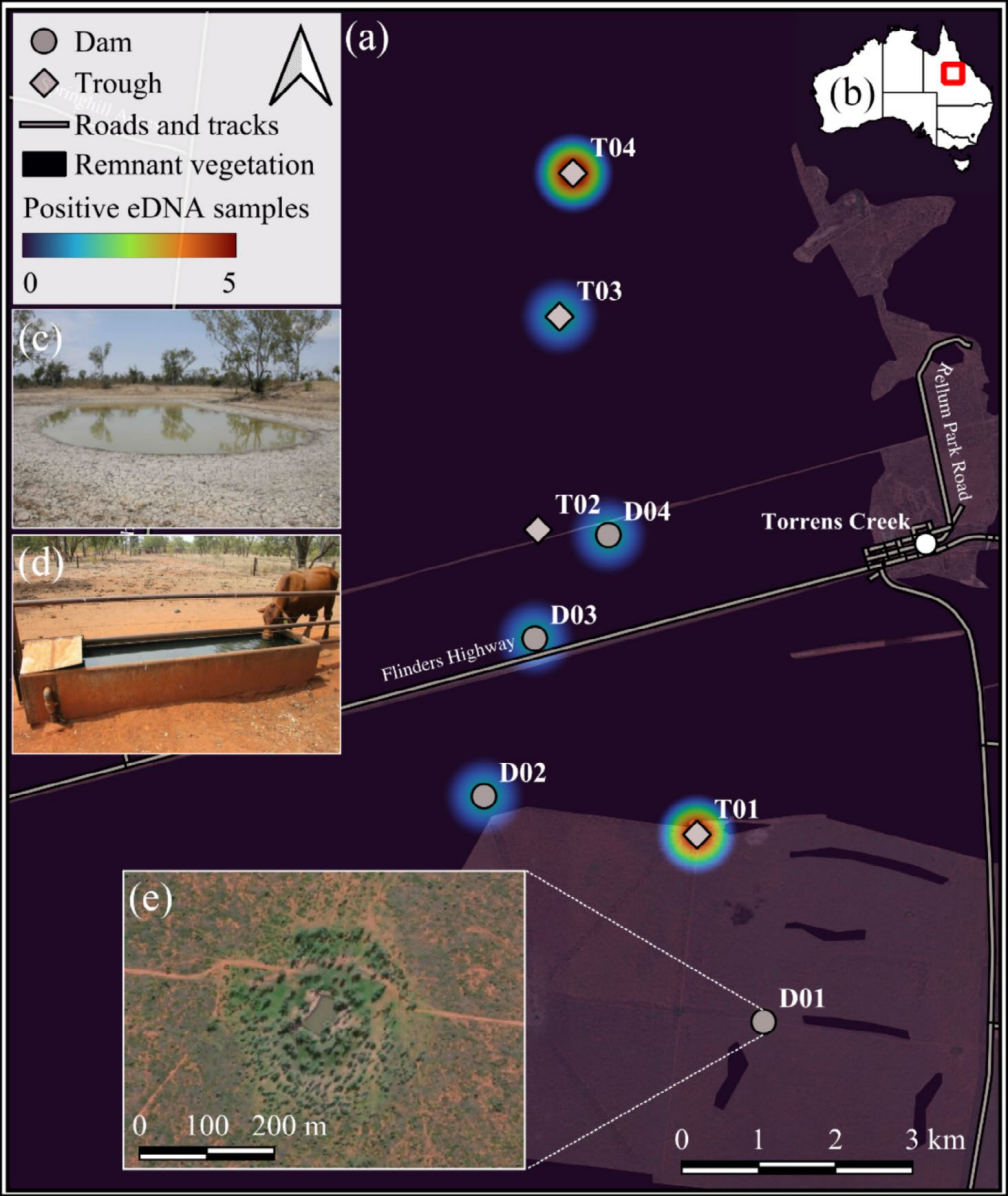
Study sites and location of (a) the eight sampled artificial waterpoints at the cattle property near Torrens Creek including the number of positive eDNA samples, delineation of 2021 remnant vegetation cover (Queensland Herbarium 2021) and (b) location within Queensland, Australia. Examples of the two types of artificial waterpoints we sampled: (c) dam and (d) water trough. The satellite imagery of (e) site D01 shows that despite being mapped as non‐remnant vegetation, there is extensive regrowth resembling pre‐cleared conditions as observed in the field.

eDNA field sampling occurred in July and November 2023 at eight waterpoints across a large beef cattle property in a semi‐arid tropical savanna near the remote township of Torrens Creek, approximately 300 km west of Townsville in northern Queensland, Australia (Figure 1). Torrens Creek has a population of less than 50 people, annual mean minimum and maximum temperatures of 17.6°C and 32.4°C, respectively, and an average annual rainfall of 452 mm (www.bom.gov.au). The region is monsoonal, with most rainfall occurring during the hotter (wet season) months between December and April. Thus, we sampled once during the early dry season (July) when temperatures were relatively cool and surface water was readily available and again in the late dry season (November) when temperatures were hot and artificial waterpoints were the primary sources of water available in the landscape. On the days of sampling, the minimum and maximum temperature was 13.9°C and 26.5°C in July and 18.4°C and 41.2°C in November. No rainfall had occurred for several weeks prior to both sampling periods.

The surrounding landscape is primarily pastoral properties with negligible urban and residential development. Vegetation communities comprise *Eucalyptus* woodlands to open woodlands on sand plains dominated by *E. whitei* (White's ironbark) or *E. similis* (Queensland yellowjacket) (Neldner et al. 2023). The understorey consists of sparse *Acacia* spp., *Carrisa* spp. or *Bursaria* spp. in the shrub layer and a ground layer dominated by *Triodia pungens* and various tussock grasses such as *Themeda* spp. or *Heteropogon* spp. Trees to the south of the property had been cleared prior to our study (Figure 1). The southern squatter pigeon has been observed across the entire study area (20,000 ha) and at sampled waterpoints for over 12 months prior to eDNA sampling via a concurrent camera trap study (data not shown). The study area is outside the range of the northern subspecies.

**FIGURE 2:**
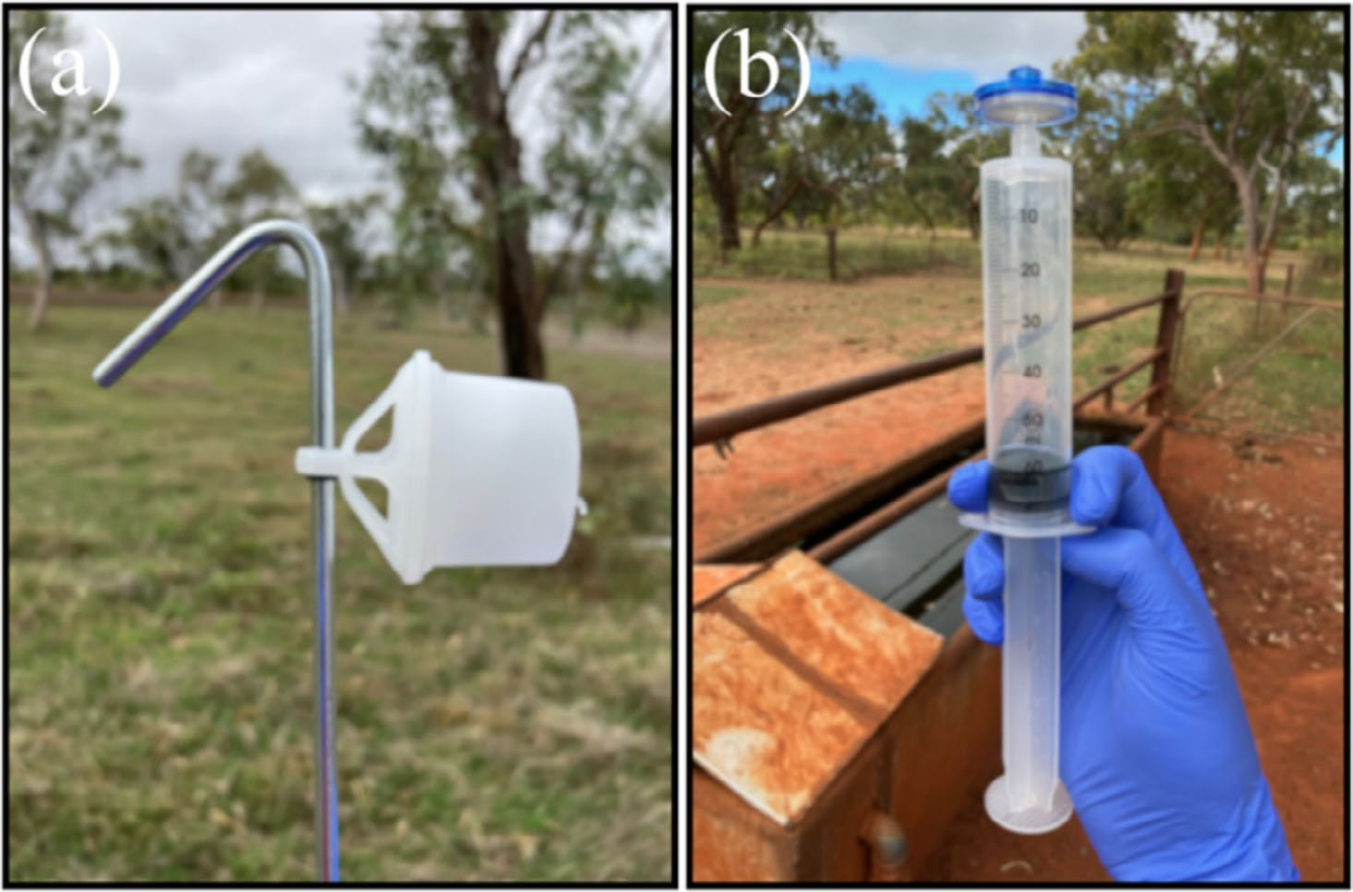
The two types of eDNA water samplers used during the study: (a) peg‐mount passive filter (WilderLab NZ Ltd) and (b) active syringe membrane filters (WilderLab NZ Ltd).

eDNA samples were collected from four randomly selected water troughs and four randomly selected dams within the cattle property in July 2023 and again in November 2023. At each site, two water samples were collected using passive filters (peg‐mount, Wilderlab NZ Ltd) and an additional two water samples were actively pushed through a 1.2 μm cellulose acetate filter membrane (BioPure) using a syringe (Figure 2). On day one, two passive filters were placed in situ in each trough and dam and left for 24 h. On day two, water was actively syringe‐filtered through filter membranes before passive filters were then collected to prevent cross‐contamination between the filter types. Approximately 1 mL of preservative (ZYMO DNA/RNA shield) was added to each filter. Two syringe‐filtered negative field control samples were included on each sampling occasion by filtering 1 L of UV‐sterilised water.

Visual surveys for squatter pigeons were undertaken at each artificial waterpoint within a 2‐ha search area over a 20‐min period. A single experienced observer (GY) recorded all pigeon sightings at the time of each eDNA sampling method (active and passive), repeated in July and November 2023 (Table 2).

**TABLE 2:** Environmental DNA samples collected across all sample sites in July and November 2023. Sites labelled D and T indicate dams and troughs, respectively. At each site, two eDNA samples of each filtration type were collected. Positive eDNA detections are underlined. Visual sightings are the number of squatter pigeons observed at each site at the time of each eDNA sampling method, passive or syringe. The volume filtered is the average of the two active syringe samples collected per site.

Month	Site	Sampling day	Filtration type	Positive eDNA samples	Volume filtered (mL)	Visual sightings
July	D01	Day 0	Passive	0/2		5
		Day 1	Syringe	0/2	150	0
July	D02	Day 0	Passive	1/2		0
		Day 1	Syringe	0/2	75	0
July	D03	Day 0	Passive	0/2		0
		Day 1	Syringe	0/2	203	0
July	D04	Day 0	Passive	0/2		0
		Day 1	Syringe	0/2	58	0
July	T01	Day 0	Passive	0/2		3
		Day 1	Syringe	0/2	240	0
July	T02	Day 0	Passive	0/2		0
		Day 1	Syringe	0/2	210	0
July	T03	Day 0	Passive	0/2		0
		Day 1	Syringe	0/2	65	0
July	T04	Day 0	Passive	1/2		0
		Day 1	Syringe	2/2	280	0
November	D01	Day 0	Passive	0/2		0
		Day 1	Syringe	0/2	265	0
November	D02	Day 0	Passive	0/2		0
		Day 1	Syringe	0/2	58	0
November	D03	Day 0	Passive	0/2		0
		Day 1	Syringe	1/2	175	0
November	D04	Day 0	Passive	0/2		1
		Day 1	Syringe	1/2	105	4
November	T01	Day 0	Passive	1/2		0
		Day 1	Syringe	2/2	110	0
November	T02	Day 0	Passive	0/2		0
		Day 1	Syringe	0/2	90	2
November	T03	Day 0	Passive	0/2		2
		Day 1	Syringe	1/2	208	0
November	T04	Day 0	Passive	0/2		4
		Day 1	Syringe	0/2	238	0

### 2.4 eDNA Extraction

All eDNA samples were extracted in a designated PCR‐free, trace DNA laboratory at the University of Canberra, using the Qiagen DNeasy Blood and Tissue kit (Qiagen Pty Ltd). An extraction negative was included with each batch of extractions. qPCRs on eDNA extracts were run in triplicate at both neat and 1:10 dilutions (to account for possible inhibition) with qPCR mastermix and cycling conditions as described above. Six no‐template control (NTC) samples and 15 positive control samples were included with each PCR plate. A replicate was considered to contain a positive amplicon if an exponential amplification curve had a *C*_t_ value less than or equal to 40 and a melt curve within Tm 0.5 of the target. To confirm amplification of the target species, a subset of positive PCR amplicons spanning four different sites was purified and sent to the Biomedical Research Facility at the Australian National University for Sanger sequencing on an ABI 3730xl DNA Analyser using the species‐specific primers.

## 3 Results

### 3.1 qPCR Assay Design

Sequence information was aligned for seven bird species including the target and co‐occurring Columbidae species (Table 1). A 135 bp region was targeted for a species‐specific qPCR assay using newly designed primers; *G. scripta_2F 5*′*‐ATCGCCTTAACCCCGCTAAC‐3*′ and *G. scripta_2R 5*′*‐GCTAAATCCGCCTTCCAGGA‐3*′. The primers provided a perfect sequence match to the target sub‐species, *G. s. scripta*, and contained a single base‐pair mismatch to the northern subspecies, *G. s. peninsulae*. The primers contained at least 4 bp mismatches to all other co‐occurring species. All secondary structures and dimers occurred within the tolerable range.

### 3.2 qPCR Assay Sensitivity and Specificity

The squatter pigeon sensitivity plate detected DNA concentrations of the target *G. s. scripta* as low as 1 × 10^−7^ ng/μL, with an efficiency of 94.1% and *r* ^2^ values of 0.992 (Figure S1). The squatter pigeon assay provided positive amplification against all target *G. s. scripta* samples tested. Positive amplification was also obtained for *G. s. peninsulae*, although *C*_t_ values were much higher, indicating reduced efficiency to detect this northern sub‐species. Internal sequence variation enabled the two sub‐species to be accurately identified by Sanger sequencing positive amplicons. All other species failed to amplify, confirming the specificity of this assay to detect the target species.

### 3.3 eDNA Sampling

Across all eight sites, we collected 64 filter samples in total over the two sampling periods. Of these, 32 were active (mean volume filtered 158 mL; ±14 SE; range 50–300 mL) and 32 via passive filtration (mean 24 h submerged; range 23–25 h) (Table 2). qPCR assays detected squatter pigeons in 10 eDNA samples, spanning both dam and trough sites across both early and late dry season (Figure 1; Table 2). All Sanger sequenced amplicons confirmed successful amplification of the target *G. s. scripta* and all field controls, extraction controls, NTCs and positive control samples performed as expected.

Squatter pigeons were visually observed on seven occasions, spanning both dam and trough sites across both seasons (Table 2). These visual observations coincided with positive eDNA detections of squatter pigeons on only one occasion (site D04 November Day 1) whilst two observations occurred the day prior to positive eDNA syringe samples (site T03 November Day 0 and site D04 November Day 0).

## 4 Discussion

We developed a novel, highly sensitive qPCR assay to detect DNA of the Vulnerable southern squatter pigeon, *G. s. scripta*, at concentrations as low as 1 × 10^−7^ ng/μL (Figure S1). This assay can be applied to facilitate detection of eDNA samples of the target species from various substrates including air, soil or water. The assay's effectiveness was validated through both active and passive water sampling methods at both farm dams and water troughs during the Australian tropical dry season in a semi‐arid tropical savanna. This is the first study known to apply passive filtration to a single species in a terrestrial habitat and only the second in Australia to design a species‐specific assay targeting a terrestrial bird, following the study by Day et al. (2019) on the Gouldian finch ( *Chloebia gouldiae* ). Although designed specifically for the southern squatter pigeon subspecies, the assay also detected the northern subspecies, *G. s. peninsulae*, due to its close genetic similarity. Internal sequence variation between the two subspecies, however, enables clear differentiation in areas of north Queensland where they co‐occur (Ford 1986).

Positive detections from both eDNA and visual surveys were scattered across sampling timepoints, waterbody type (i.e., farm dams and water troughs) and, for eDNA, across both active and passive sample types. The two survey methods rarely detected the species at the same site, highlighting the complementary nature of eDNA and visual detection methods. Overall detection rates were similar, with both eDNA and visual surveys identifying the target species at 75% of the sites (Table 2). However, visual surveys may offer greater practical value due to their lower cost and ability to provide immediate confirmation of species presence. Consequently, visual surveys may still be the easiest detection method for *G. s. scripta*, at least in locations where the species is relatively abundant. eDNA sampling could complement such conventional methods in areas where the birds are increasingly difficult to locate, such as southern Queensland and New South Wales.

eDNA has proven valuable for enhancing detection of avian and mammalian communities when used in conjunction with camera trapping (Tetzlaff et al. 2024) or radio‐tracking for post‐release monitoring of reptiles (Nordstrom et al. 2024). Under suitable environmental conditions, DNA residues can persist in water for up to 2 weeks (Barnes et al. 2014). In the semi‐arid environment encountered at our study site, however, high ambient temperatures and UV exposure are likely to accelerate DNA degradation (Tsuji et al. 2017). Indeed, Day et al. (2019) showed eDNA degraded in less than 12 h in water exposed to the sun in a similar climate in the Northern Territory, Australia. High concentrations of algae and bacteria, likely to be present at the artificial water points encountered here, are also likely to further accelerate DNA decay (Barnes et al. 2014; Joseph et al. 2022). This rapid eDNA degradation and/or dispersion may account for several instances where squatter pigeons were visually confirmed on Day 0 but failed to be detected by eDNA sampling the following day (Table 2) (Furlan et al. 2016; Strickler et al. 2015). Alternatively—or in addition—failure to detect squatter pigeon eDNA at visually confirmed sites may reflect an individual's limited interaction with the water source or insufficient DNA shedding to enable eDNA detection.

Both the passive and active eDNA filtration methods detected the target species at water sampled from both farm dams and cattle drinking troughs. Further research may determine whether one eDNA sampling approach is more effective than the other. Although our limited sampling prohibits statistical comparison, we did achieve more positive eDNA detections through active eDNA sampling (Table 2). The lack of natural water flow at both dams and troughs likely reduced the capacity of passive samplers to filter sufficient volumes of water to effectively capture eDNA—these sample types may perform best in flowing rivers or streams (Chen et al. 2024). High sediment concentrations in artificial waterpoints may also restrict the volume of water filtered as well as increase inhibition, hindering the efficiency of eDNA surveys (Furlan et al. 2019; Saeki and Sakai 2009). Active sampling filters clogged rapidly, limiting the volume of water that could be sampled to between 58 and 280 mL (Table 2). High turbidity at dam sites, in particular, limited sampling volumes (average 136 mL compared to 180 mL at troughs), which may have contributed to a lower number of eDNA detections at these locations (Table 2). Future research would benefit from thoroughly evaluating detection probabilities of these two eDNA sampling methods across different artificial waterbodies to optimise future eDNA surveys.

Varying active or passive sampling methods may also prove valuable to increase the efficiency of eDNA surveys. For example, varying filter pore sizes to increase water volume and maximise eDNA capture (i.e., Bird et al. 2024) may overcome the challenges associated with turbid artificial waterpoints. Meanwhile, the period of time that passive filters remain in contact with the water may vary detection probabilities: prolonging exposure may extend the window of detectability for species that visit and deposit DNA intermittently and may offer an advantage in hot environments where DNA degrades rapidly (Jo et al. 2019; Strickler et al. 2015; Tsuji et al. 2017). Contrastingly, Bessey et al. (2022) showed a submersion time of just 5 min was all that was required to achieve species richness and qPCR detections similar to active filtration methods in an aquarium, whilst a passive sweep approach (sweeping the filter through the water for several seconds) has been shown to detect a diverse range of taxa (McDonald et al. 2023). Despite passive filtration for species‐specific detection primarily being limited to laboratory (Chen et al. 2022) and mesocosm (Bessey et al. 2022) environments, new techniques are continuously emerging, each with their own enhanced ability for eDNA adsorption (see Jeunen et al. 2022; Verdier et al. 2022). Hence, continued testing is required to determine more appropriate sampling methods at artificial waterpoints along with experimental designs that generate the most reliable inference (De Brauwer et al. 2022). Logistical considerations also need to be considered and may dictate the optimal eDNA sampling approach in remote locations. Active sampling, despite requiring more time on‐site to filter samples, avoids the need for a return visit to retrieve samples. In contrast, passive filters are less labour‐intensive, can be rapidly deployed and retrieved or left in place for extended periods (i.e., 24 h of continuous sampling), allowing for a longer sampling duration (Bessey et al. 2021).

Investigating alternate sampling approaches may also help increase eDNA detectability. Leveraging a deeper understanding of the ecology of the target species will likely lead to better targeted DNA sampling. For example, *G. scripta* prefer to drink from the water's edge and are likely to have limited interaction with a larger water source. Consequently, their DNA might be more abundant around the edges of water sources, having shed from their mouth, faeces, feet or other skin cells deposited near the water's edge. Alexander et al. (2023) and Jarman et al. (2024) successfully scraped or swabbed semi‐submerged man‐made marine infrastructure to detect invasive marine species—an approach which might be adaptable for detecting pigeons or other terrestrial birds using artificial waterpoints. eDNA detectability may also be increased by selecting naturally shaded sites or introducing artificial shading to increase the persistence time of DNA (Day et al. 2019). Designing attractants to lure birds into smaller and cleaner bodies of water (e.g., establishment of bird baths) will likely reduce issues of inhibition and sedimentation, leading to larger sample volumes of DNA and reduce the presence of algae or bacteria, leading to increased DNA persistence times (Barnes et al. 2014; Joseph et al. 2022). Moreover, increasing the sampling intensity (e.g., more replicates, more frequently) will also enhance eDNA detectability (Furlan et al. 2016). Nonetheless, despite sampling very low volumes of water across a limited number of replicates (Table 2), we were still able to successfully detect the squatter pigeon across multiple sites from as little as 105 mL of water, highlighting the sensitivity of our DNA assay to detect the species.

## 5 Conclusions

The use of eDNA as a non‐invasive and highly sensitive detection tool has gained significant popularity over the past decade, particularly in aquatic environments. However, its application in monitoring terrestrial species, especially in arid or semi‐arid environments, is still emerging. Artificial waterbodies are abundant in agricultural landscapes and provide a crucial resource for water‐dependent species in arid and semi‐arid regions. Their importance in these environments makes them ideal locations for conducting eDNA surveys, acting as a natural DNA sink at which to target detection efforts. In this study, we developed a species‐specific qPCR assay to detect the southern squatter pigeon, providing an additional tool to assist in the conservation, monitoring and management of this species. We have demonstrated the potential for eDNA surveys to detect these ground‐nesting birds at artificial waterbodies from both dams and water troughs. eDNA surveys at these sites could similarly benefit other species including numerous birds, amphibians, reptiles, mammals and invertebrates that interact with these artificial water sources. By enhancing our knowledge of species distributions and improving monitoring efforts, eDNA surveys at artificial waterbodies can play a crucial role in future conservation efforts of species in remote, arid environments.

## Author Contributions

**Gary Young:** conceptualization (lead), data curation (equal), formal analysis (equal), funding acquisition (equal), investigation (equal), methodology (equal), software (equal), visualization (equal), writing – original draft (lead). **Benjamin L. Allen:** conceptualization (equal), funding acquisition (equal), investigation (equal), methodology (equal), supervision (equal), writing – review and editing (equal). **Peter J. Murray:** conceptualization (equal), funding acquisition (equal), investigation (equal), supervision (equal), writing – review and editing (equal). **Elise M. Furlan:** data curation (equal), formal analysis (equal), investigation (equal), methodology (equal), software (equal), supervision (lead), visualization (equal), writing – original draft (equal), writing – review and editing (equal).

## Ethics Statement

An exemption was given to the authors from requiring an animal ethics permit as no work was undertaken with live animals (Ref: 21EXE009).

## Conflicts of Interest

The authors declare no conflicts of interest.

## Supporting information


**Figure S1** The amplification efficiency and sensitivity achieved of the *Geophaps scripta* qPCR assay developed in this study.

## Data Availability

All data used can be found within the report. The sequence data from tissue samples of the target species are available in GenBank (accession numbers PQ824958–PQ824961) at the National Center for Biotechnology Information.
