# Supplementary material for: Artificial Waterbodies: A Valuable Source of eDNA for Detecting Threatened Birds
Source: Ecol Evol. 2025 Jun 5;15(6):e71509. doi: 10.1002/ece3.71509 (PMC12138456; doi:10.1002/ece3.71509)

**APPENDIX**

**Figure S1:** The amplification efficiency and sensitivity achieved of the *Geophaps scripta scripta* qPCR assay developed in this study.


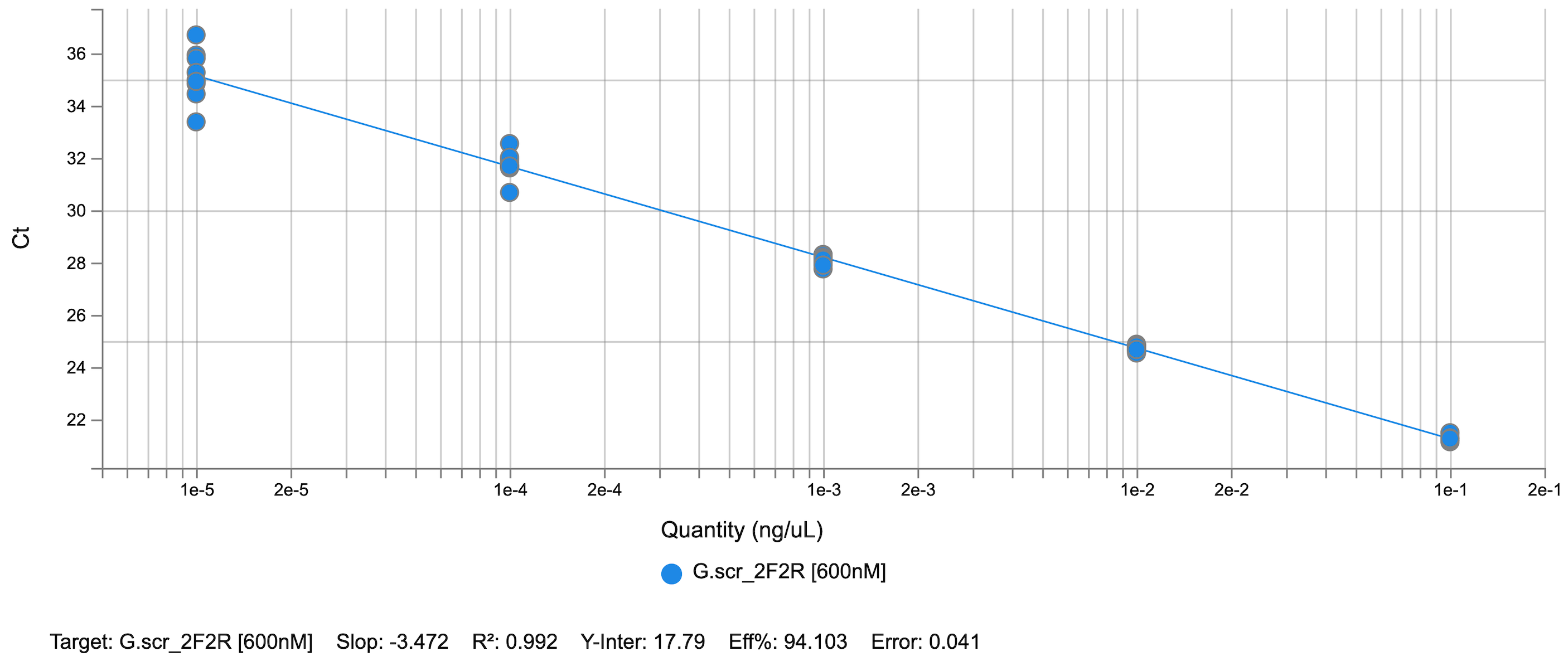

Supplement: Supplementary file 1 — Figure S1 The amplification efficiency and sensitivity achieved of the Geophaps scripta qPCR assay developed in this study. [file ECE3-15-e71509-s001.docx]
